# Microbiological and Chemical Quality of Portuguese Lettuce—Results of a Case Study

**DOI:** 10.3390/foods9091274

**Published:** 2020-09-11

**Authors:** Catarina Ferreira, Filipa Lopes, Reginaldo Costa, Norton Komora, Vânia Ferreira, Virgínia Cruz Fernandes, Cristina Delerue-Matos, Paula Teixeira

**Affiliations:** 1 CBQF—Centro de Biotecnologia e Química Fina—Laboratório Associado, Escola Superior de Biotecnologia, Universidade Católica Portuguesa, Rua Diogo Botelho 1327, 4169-005 Porto, Portugal; catarinarpferreira@gmail.com (C.F.); pipalopes96@gmail.com (F.L.); reginaldo05_@hotmail.com (R.C.); nortonsnk@hotmail.com (N.K.); vferreira@porto.ucp.pt (V.F.); 2 REQUIMTE/LAQV, Instituto Superior de Engenharia do Porto, Instituto Politécnico do Porto, Rua Dr. António Bernardino de Almeida, 431, 4200-072 Porto, Portugal; vircru@gmail.com (V.C.F.); cmm@isep.ipp.pt (C.D.-M.)

**Keywords:** organic produce, microbiological contamination, chemical contamination, foodborne pathogens, pesticide residues

## Abstract

In addition to environmental pollution issues, social concerns about the sustainability, safety, and quality of conventionally grown fruits and vegetables have been increasing. In order to evaluate if there were any microbiological differences between samples of organic and conventional lettuce, a wide range of parameters were tested, including pathogens and indicator organisms: the enumeration of *Escherichia coli*; the detection of *Salmonella* spp.; the detection/enumeration of *Listeria monocytogenes*; the enumeration of lactic acid bacteria, *Pseudomonas* spp. yeasts and molds, and *Enterobacteriaceae*. This study also evaluated the chemical safety of the lettuce samples, quantifying the nitrate concentration and 20 pesticides (14 organochlorine and 6 organophosphorus pesticides). Significant differences (*p* < 0.05) between the conventional and organic samples were only detected for the counts of total microorganisms at 30 °C. Pathogens were absent in all the samples. The analytical method, using the quick, easy, cheap, effective, rugged, and safe (QuEChERS) approach for pesticide extraction, was suitable for detecting the targeted analytes; the limit of quantification (LOQ) was between 0.6 and 1.8 µg/kg (lower than the Maximum Residue Levels (MRLs) established by EU legislation). In three organic lettuce samples, one organochlorine pesticide (α-HCH) was observed below the MRLs. For the samples analyzed and for the parameters investigated, except for the total mesophilic counts, the organic and conventional lettuces were not different.

## 1. Introduction

“Fruits and vegetables are important components of a healthy diet”, and their daily consumption reduces the risk of severe illnesses, such as cardiovascular diseases and certain types of cancer [1,2]. Despite these scientifically validated health benefits, contaminated produce (by virus, bacteria, or parasites) has been linked to major cases and outbreaks of foodborne diseases in recent years, and has led to some of the biggest food recalls [3,4]. Among the top 10 multistate outbreaks ranked by the number of illnesses in 2019 in the USA, six were linked to the consumption of raw produce [5]. In addition to biological threats, contamination by chemicals from anthropogenic and natural sources is another global food safety issue [6]. These chemicals belong to several groups, with pesticides receiving the most attention. The consumption of pesticide-contaminated food via one’s daily diet is a major source of exposure to pesticides, which may pose adverse effects to humans. leading to acute, chronic, or subchronic problems [7]. Some fruit and vegetables are at the top of the list of foods that contain the highest pesticide levels, since most of them are grown close to the soil, are eaten raw, and are without the need to remove the skin [8]. Lettuce is one of the most consumed leafy vegetables worldwide, being a basic component of raw salads prepared in domestic households. Lettuce was included in the last dirty list of fruit and vegetables [9], and in the last EU report, it was one of the commodities that were found to contain multiples residues in more than 50% of the samples analyzed [10]. In addition, lettuce has been implicated in reported outbreaks of microbial foodborne diseases [4]. 

During the last years, social concerns with the sustainability, safety, and quality of conventionally grown fruits and vegetables have increased, in addition to environmental pollution issues which cause ecosystem disequilibrium [11]. This has led to a growing recognition of the need to develop alternative agricultural practices, such as biological/organic production [12]. 

The consumption trend of organic food has grown, and it is becoming one of the most valuable market segments in the food industry. Consumers are willing to pay more for this type of product [11,13], believing that they are safer, healthier, and nutritionally richer than conventional ones [11,13,14].

Despite the strong belief that organic foods are safer than conventional foods, this has not to date been validated by scientific research [15]. Regarding sensory attributes (visual aspect, taste, texture, bitterness, and overall liking), in a recent study conducted by da Cunha et al. [16] the participants did not perceive differences between conventional and organic leafy greens in blind tests. However, organic products received higher scores in the informed test and in the inverted test (conventional labelled as organic). 

Regarding organic farming, the organic food produced should fit into the Regulation (EC) No 2018/848. Nevertheless, no specific Maximum Residue Levels (MRLs) are established for these organic products, and the same MRLs ((EC) No. 396/2005) [17] as conventional food are used. The 2019 European Food Safety Authority (EFSA) report on pesticide residues in food [18] concluded that there is a lack of work in the scientific literature on organic products. There are only a few recent studies that describe chemical safety in terms of pesticides in organic lettuce samples [19,20,21]. Similarly, and in accordance with the European food legislation (Regulation (EC) No. 2073/2005), the same microbiological criteria were established for conventional and organic foods. As for pesticides, only a few studies have been recently conducted regarding the microbiological safety of organic lettuce samples [22,23,24]. 

The main objective of this study was to compare organic and conventional lettuce (*Lactuca sativa*) samples regarding their microbiological characteristics, nitrate concentrations, and levels of pesticide residues. Despite the low number of samples analyzed, to our knowledge such a comprehensive and comparative study has not been developed in recent years. Such information is a prerequisite for a conscious choice of foods and even lifestyles by the citizens, and therefore needs to be extended to a higher number of samples and to different products.

## 2. Materials and Methods

### 2.1. Sampling

Ten lettuces from certified organic production and 10 from conventional production were purchased in supermarkets or farmers’ markets located in Porto between 11 March and 6 May 2019. All the samples were from Romaine lettuces grown in Portugal. Whenever possible, at each location and date of purchase the same number of biological and conventional samples were purchased. Samples were transported to the laboratory at room temperature and analyzed in less than 24 h after being collected. 

For chemical safety analysis, the samples were separately frozen at −18 °C. Each sample was cold-homogenized using a high-performance blender (Vorwerk, Portugal). The samples were then stored at −20 °C. The homogenized samples were stored in the freezer before being thawed just prior to extraction.

### 2.2. Microbiological Analysis

Twenty-five grams samples of each lettuce (unwashed samples) were added to 225 mL of sterile buffered peptone water and homogenized in a Stomacher BagMixer (Interscience, Saint Nom la Brèteche, France) for 1 min, set to velocity 3. Appropriate decimal dilutions were prepared in Ringer’s solution for the microbial enumeration of lactic acid bacteria [25], *Enterobacteriaceae* [26], *Escherichia coli* [27], *Listeria monocytogenes* [28], yeasts and molds [29], total counts at 30 °C [30], and *Pseudomonas* spp. [31]. The detection of *L. monocytogenes* [32] and *Salmonella* spp. [33] was also performed.

### 2.3. Chemical Safety Analysis

#### 2.3.1. Chemicals, Materials and Standards

The chromatographic-grade acetonitrile (MeCN) was from Carlo Erba (Val de Reuil, France), and the n-hexane and sodium acetate were from Merck (Darmstadt, Germany). The lead acetate and lead oxide were from Sigma (Steinheim, Germany).

All 20 pesticides (14 organochlorine pesticides (OCP) (α-, β-, γ-, and ζ- hexachlorocyclohexanes (HCH); hexachlorobenzene (HCB); [1,1,1 trichloro-2,2-bis- (p-chlorophenyl) ethane] (o,p′-DDT); [2,2-bis(p-chlorophenyl)-1,1-dichloroethylene] (p,p′-DDE); 1-chloro-4-[2,2-dichloro-1-(4-chlorophenyl)ethyl]benzene (p,p′-DDD); aldrin; dieldrin; endrin; α-, β-endosulfan; and methoxychlor) and six organophosphorus pesticides (OPP) (chlorfenvinphos, chlorpyrifos, chlorpyrifos methyl, diazinon, malathion and parathion methyl) were acquired from Sigma Aldrich (St. Louis, MO, USA), Chemservice (West Chester, PA, USA), Dr. Ehrenstorfer GmbH (Augsburg, Germany)) were at analytical grade (purity above 97%). 4,4′-dichlorobenzophone and triphenyl phosphate (TPP), used as internal standards (IS) for OCP and OPP, respectively, were from Sigma Aldrich (St. Louis, MO, USA). Original quick, easy, cheap, effective, rugged, and safe (QuEChERS) and cleanup (with 150 mg of MgSO4, 50 mg of PSA, and 50 mg of carbon) were purchased from Agilent Technologies (Santa Clara, CA, USA). Stock mixture solutions, one with 14 OCP and other with six OPP, were prepared at 2000 µg/L in n-hexane and stored at −18 °C. From these stock solutions, two standard mixtures containing 14 OCPs and another with 7 OPP were prepared in n-hexane at 150 µg/L. The Internal Standards (IS) were prepared in n-hexane at 2000 µg/L. For pesticide determination, various concentrations were used for the preparation of seven matrix-matched standard calibration solutions (between 3–150 µg/kg) and the recovery studies at three spiking levels (25, 50, 100 µg/kg) by the dilution of stock solutions with a matrix extract. The IS solutions were used in all experiments.

#### 2.3.2. Nitrate Concentration

The determination of nitrate concentration in lettuce samples was performed according to the potentiometric method described by Lima et al. [34], with some modifications. Briefly, two grams of fresh lettuce leaves were dried (100 °C) and powdered; then, approximately 50 mg of lettuce powder was mixed with the 10 mL of nitrate extraction solution (ISA-Pb) composed of 1 × 10^−2^ M lead acetate, 5 × 10^−3^ M lead oxide, and 1 × 10^−2^ M sodium acetate; shaken for 5 min; and filtered. Determinations were performed over this extract solution in a nitrate ion-selective electrode. This was the method performed because its efficiency had already been proved, as well as cost-effectiveness and quickness. 

#### 2.3.3. Pesticides Determination 

##### Sample Preparation

**Scheme 1:**
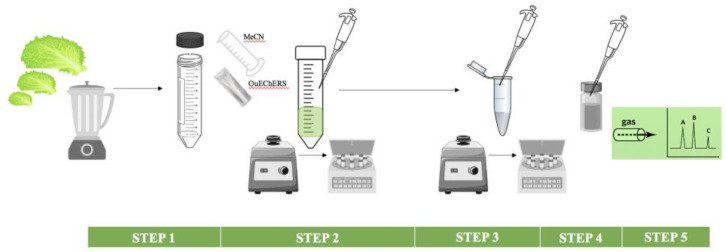
Schematic illustration of the extraction procedure.

The OCP and OPP were extracted from the lettuce samples based on the previously reported QuEChERS approach with dispersive solid-phase extraction (d-SPE) cleanup [35]. A schematic illustration of the method is shown in Scheme 1 and included the five steps: (1) 5.0 g of a homogenized sample or spiked sample was weighed into a 50 mL polypropylene tube, 10 mL of MeCN was added, and the tube was directly vortexed; (2) original QuEChERS powders (4 g of anhydrous MgSO_4_ and 1 g of NaCl) were added, the tubes were vortexed for 1 min, and centrifuged for 5 min at 4500 rpm at room temperature; (3) 1 mL of the upper layer was transferred to the d-SPE cleanup tube (150 mg of MgSO_4_, 50 mg of PSA, and 50 mg of carbon), and the tubes were vortexed for 5 min at 4500 rpm at room temperature; (4) 800 µL of the cleaned extract was transferred to a labeled vial, the extract was dried by exposing it to a low flow of nitrogen gas, and it was re-dissolved in n-hexane; finally, (5) the sample extract was vortexed and 2 μL of the extract was used for the gas chromatogrphy (GC) analysis.

##### Method Validation

For analytical validation, the following parameters were evaluated: the linearity in solvent and matrix, the recoveries, the limit of detection (LOD), the limit of quantification (LOQ), and the method accuracy and precision. A matrix blank extract was used for the preparation of a set of experiments—namely, 7 matrix-matched calibration standards (3–150 µg/kg) and three sets of matrix spiking at low, medium, and high levels (25; 50; 100 µg/kg) for all pesticides. Precision was calculated in terms of the intraday repeatability RSD% (*n* = 3) to three spiking levels for all the analytes.

##### GC Analysis

The gas chromatographic analysis was performed using three different types of equipment, (Table 1) including an electron capture detector (ECD), a flame photometric detector (FPD), and a mass spectrometry detector (MS). For the confirmation of the positive samples, GC/MS in Selected Ion Monitoring (SIM) mode was performed. The National Institute of Standards and Technology (NIST) and Wiley pesticide libraries were used for the selection of the selected ions m/z. The instrumental and operational parameters used for the analysis of OCP and OPP are summarized in Table 1. 

### 2.4. Statistical Analyses

To establish a comparison between organic and conventional products regarding the microbiology parameters and nitrate concentration, the results were analyzed using a one-way analysis of variance (ANOVA) with Tukey’s post-hoc test (SPSS, Version 23.0, Inc., Chicago, IL, USA). 

## 3. Results and Discussion

### 3.1. Microbiological Analysis

The results of the microbiological analyses are shown in Table 2. In general, variability was observed between samples. For most of the parameters analyzed, the mean counts were higher for conventional than for organic samples, but with the exception of total counts at 30 °C, no other differences were statistically significant (*p* > 0.05). In general, the microbial counts observed in the present study were higher than those previously reported [22,24,36]. It should be highlighted that, while in the present study, samples were purchased without any indication of collection dates because they were unpacked at the point of purchase, in the study by Oliveira et al. [22] the samples were collected directly from farms.

*Salmonella* spp. and *L. monocytogenes* were absent in all the samples analyzed; *E. coli* was always below the detection limit of the enumeration technique. These pathogens have been occasionally found in lettuce samples from organic and conventional origin [22,24,36]. As in previous studies [22,24], no differences between organic and conventional lettuce were observed regarding the microbiological safety. It is important to highlight that most samples were purchased in stores of large supermarket chains with stringent food quality and food safety standards.

### 3.2. Nitrate Concentration

There were no significant differences (*p* > 0.05) between the nitrate content of organic and conventional lettuce (Table 3). Although we do not have information on the growth conditions of the samples analyzed (e.g., fertilization regimes), the fact that all the samples were of the same variety and were probably grown under similar light conditions may justify this observation [37]. According to the EFSA [38], the maximum levels for nitrate in fresh lettuce (*Lactuca sativa*) are between 2500 and 4500 mg/kg. It was possible to observe that, in organic lettuce, the nitrate values varied between 1400 and 1700 mg/kg and, on the other hand, the values in conventional lettuce varied between 1200 and 1900 mg/kg, which means that all the samples are within the recommended limits. According to Laia et al. [39], in Portugal the values of nitrate concentration for lettuce samples through the year of 2018 varied between 528 and 2369 mg/kg, meaning that our results are within the same range.

### 3.3. Pesticide Analysis

#### 3.3.1. Method Evaluation

The analytical methodology was validated according to the EU Commission’s DG Health & Food Safety (SANTE) guidelines [40]. Table 4 showed a summary of the analytical validation parameters. Suitable matrix-matched calibration curves were obtained. Appropriate coefficients of determination (*R*^2^) were obtained for the scope of the targeted pesticides, with *R*^2^ in the range of 0.9900–0.9994. The LOQ values were similar to or better than those of previously published works [19,20,21]. The LOQ ranged from 0.77 to 1.79 µg/kg for OCP and 0.57–1.57 µg/kg for OPP. The LOQ values are lower than the MRLs set by the European Commission (shown in Table 4). The recoveries of the method were evaluated at three concentration spiking levels, and were within acceptable ranges as set by European Union SANCO guidelines (70–120%). Except for malathion, which presented a recovery percentage of between 55% and 65%, the remaining pesticides showed values of between 72% and 97%, and with suitable precision and relative standard deviation (RSD) values below 20%. These results demonstrated that the developed method had a good analytical performance to apply in food safety analysis. 

#### 3.3.2. Sample Analysis

The validated method was applied to determinate the pesticide residues in 10 conventional lettuce and 10 organic lettuce samples. Only one organochlorine pesticide (α-HCH) was detected in three of the organic farming lettuce samples analyzed at a concentration of between (Org7 and Org9) and 0.11 µg/kg (Org6). The detectable concentrations were below the MRLs established by the European Commission. Despite being banned several years ago in the developed world, levels of these pollutants have been detected by researchers in Portugal and other countries [41,42] in various matrices, including environmental [43,44], food [42,45,46], and human samples [47]. These results contribute once again to reinforce the fact that these compounds due to their chemical characteristics persist in the environment. In addition, despite the illegal use of OCP in Europe, they were not reported in Portugal, but their extensive use remains ongoing in the African continent [41]. Indeed, these compounds have been found in vegetables (tomato, cabbage, and lettuce), and the authors of this study [42] reported that the most detected OCP in a greater concentration were the total HCHs (mean ΣHCHs = 15.115 µg/kg), followed by the total DDTs (mean ΣDDTs = 0.451 µg/kg). The presence of OCP was also reported in organic-farmed strawberries [45]. Regarding OPP, no detection was observed in the studied samples. However, in recent studies, the presence of OPP (e.g., chlorpyrifos) has been reported in organic lettuce [21] and conventional strawberries [46]. The results obtained in the present study showed that there are concerns with OPP in regards to chemical safety.

## 4. Conclusions

Regardless of the type of production, no harmful organisms nor chemical hazards were found in the lettuce samples analyzed. Despite the low number of samples analyzed, which we recognize as a limitation of this study, it is important to highlight that most of the samples were purchased in big retailers that are represented all over the country. These results tend to support the idea that big retailers demand high quality and safety standards from their suppliers. 

Regarding pesticide analysis, the analytical parameters showed that the method presented a suitable analytical performance, including satisfactory LOQ, recoveries, and precision for the target analytes. The present work demonstrated that the developed method was reliable, simple, and environmentally friendly. Additionally, the method was robust and was successfully applied to detect pesticides in a total of 20 organic and conventional lettuce samples. The results were consistent with previous findings, which showed that organic foods contain few residues. It is therefore proposed to perform continuous monitoring studies of pesticide residues in vegetables, even though they are from organic production.

## Figures and Tables

**Table 1:** Summary of gas chromatographyGC conditions for the analysis of 20 pesticides.

	GC-ECD	GC-FPD	GC/MS
**Equipment**	Shimadzu GC-2010 gas chromatograph (Shimadzu, Kyoto, Japan)	Shimadzu GC-2010 Plus gas chromatograph (Shimadzu, Kyoto, Japan)	Thermo Trace-Ultra GC from Thermo Fisher Scientific (Waltham, MA, USA) coupled to an ion trap mass detector Thermo Polaris
**Column**	Zebron-5MS, 30 m × 0.25 mm, 0.25 µm film		
**Carrier Gas**	Helium at 1 mL min^−1^		
**Injection**	2 µL splitless		
**Temperature:** Injector Detector	250 °C	250 °C	250 °C
	300 °C	290 °C	Transfer line 250 °C/Ion source 270 °C
**Temperature program**	Initial 40 °C, hold 1 min, then 20 °C/min to 120 °C, hold 1 min, next 10 °C/min to 150 °C, hold 1 min, next 10 °C/min to 180 °C, hold 1 min, next 20 °C/min to 200 °C, hold 1 min, next 10 °C/min to 290 °C and hold 2 min.	Initial 100 °C, hold 1 min, then 20 °C/min to 150 °C, hold 1 min, next 2 °C/min to 180 °C, hold 2 min, and 20 °C/min to 270 °C and hold for 1 min.	Same as ECD
**Total running time**	27 min	26 min	27 min
**Others:**			SIM mode confirmation α-HCH | m/z 109, 181, 219

**Table 2:** Microbiological characteristics of organic and conventional lettuce samples.

		Log (CFU/g)					
Production Mode	Sample	Enterobacteria	Total Counts	Lactic Acid Bacteria	*Pseudomonas* Spp.	Molds	Yeasts
Conventional	Conv1	6.5	8.0	3.4	7.7	4.3	6.0
	Conv2	4.3	8.3	4.2	7.4	4.6	7.2
	Conv3	7.1	7.8	3.4	1	4.5	5.3
	Conv4	4.9	8.0	3.2	6.4	4.8	5.6
	Conv5	5.1	8.3	7.3	7.3	5.2	5.9
	Conv6	7.0	8.9	3.9	7.4	5.1	5.5
	Conv7	4.9	8.5	*	7.0	4.4	7.1
	Conv8	6.4	9.2	4.2	7.1	4.2	6.4
	Conv9	6.0	9.2	4.0	6.6	5.4	7.0
	Conv10	6.8	7.8	1.0	6.1	4.9	5.8
	Average ± SD	5.9 ± 1.0	^b^ 8.4 ± 0.54	3.8 ± 1.6	6.4 ± 2.0	4.7 ± 0.41	6.2 ± 0.7
Organic	Org1	4.7	7.8	3.4	7.6	3.4	4.3
	Org2	<1	7.2	3.5	3.7	4.6	5.2
	Org3	6.4	7.5	2.7	7.5	5.4	6.1
	Org4	3.0	7.4	1.0	7.2	4.9	9.2
	Org5	4.1	6.1	1.0	6.0	2.2	4.5
	^a^ Org6	7.5	8.9	3.3	7.3	5.5	6.3
	^a^ Org7	4.3	7.4	3.6	6.6	5.0	5.6
	^a^ Org8	6.7	7.4	3.3	6.9	5.3	6.3
	Org9	6.1	8.0	*	7.4	4.5	6.7
	Org10	6.9	8.1	1.0	6.8	5.4	6.4
	Average ± SD	5.2 ± 1.5	^b^ 7.6 ± 0.72	2.5 ± 1.2	6.7 ± 1.2	4.6 ± 1.1	6.1 ± 1.4

^a^ Purchased in farmers’markets; *: not determined; SD: standard deviation; ^b^ average values for the total counts on organic and conventional lettuce samples were significantly different (*p* < 0.05). For all the samples, the counts of *L. monocytogenes* and *E. coli* were below the detection limit of the enumeration technique (1.0 × 10^1^ CFU/g). *Salmonella* spp. and *L. monocytogenes* were not detected in any sample.

**Table 3:** Nitrate concentration in the organic and conventional lettuce samples.

Production Mode			
Conventional		Organic	
Sample	Nitrate (mg/kg)	Sample	Nitrate (mg/kg)
Conv1	1290	Org1	1550
Conv2	1760	Org2	1530
Conv3	1650	Org3	1720
Conv4	1590	Org4	1670
Conv5	1630	Org5	1410
Conv6	*	^a^ Org6	1580
Conv7	*	^a^ Org7	1610
Conv8	1950	^a^ Org8	*
Conv9	1370	Org9	1480
Conv10	1606	Org10	1569
Average ± SD	1606 ± 207		1569 ± 93

^a^ Purchased in farmers’markets; *: not determined; SD: standard deviation; average values for the nitrate concentration of organic and conventional lettuce samples were not significantly different (*p* > 0.05).

**Table 4:** Analytical performance for organochlorine (OCP) and organophophorus pesticides OPPand in lettuce matrix using the proposed method.

		Recoveries ± Relative Standard Deviation (RSD) (%) (*n* = 3)					
	Analytes	MRL * µg/kg	Coefficient of Determination	LOQ µg/kg	25 µg/kg	50 µg/kg	100 µg/kg
Organophosphorus pesticides	Diazinon	10	0.9956	1.13	78 ± 15	81 ± 18	85 ± 17
	Chlorpyrifos-methyl	10	0.9929	1.57	70 ± 12	84 ± 10	83 ± 13
	Parathion-methyl	10	0.9989	0.57	74 ± 17	95 ± 9	80 ± 15
	Malathion	500	0.9982	0.72	55 ± 20	60 ± 10	65 ± 14
	Chlorpyrifos	10	0.9963	1.03	79 ± 10	87 ± 17	90 ± 16
	Chlorfenvinphos	10	0.9956	1.13	71 ± 16	83 ± 10	79 ± 9
Organochlorine pesticides	α-HCH	10	0.9980	0.77	85 ± 16	90 ± 8	87 ± 18
	HCB	10	0.9927	1.46	90 ± 15	95 ± 15	90 ± 9
	β-HCH	10	0.9960	1.08	78 ± 11	84 ± 10	80 ± 12
	lindane (γ-HCH)	10	0.9944	1.11	89 ± 20	97 ± 11	90 ± 14
	ζ-HCH	10	0.9900	1.49	75 ± 8	82 ± 8	80 ± 1
	Aldrin	10	0.9976	0.73	79 ± 7	84 ± 7	79 ± 7
	Endosulfan I	50	0.9943	1.12	80 ± 2	89 ± 5	85 ± 4
	p.p’-DDE	50	0.9932	1.13	81 ± 2	92 ± 5	90 ± 1
	Dieldrin	10	0.9979	0.80	90 ± 5	94 ± 11	90 ± 9
	Endrin	10	0.9925	1.79	78 ± 5	86 ± 8	82 ± 4
	DDT ^#^	50	0.9958	0.96	72 ± 6	81 ± 10	80 ± 5
	p.p′-DDD	50	0.9913	1.59	75 ± 19	82 ± 11	78 ± 11
	Endosulfan II	50	0.9956	0.79	82 ± 2	90 ± 9	86 ± 1
	Methoxychlor		0.9994	0.78	73 ± 2	80 ± 7	79 ± 3

* Current Maximum Residue Levels (MRLs) obtained from European Union Pesticide Residues Database ^#^ MRL: DDT (sum of p,p′-DDT, o,p′-DDT, p-p′-DDE, and p,p′-TDE (DDD), expressed as DDT).
